# Common Diagnoses among Refugee Populations: Linked Results with Statewide Hospital Discharge Database

**DOI:** 10.29024/aogh.2354

**Published:** 2018-10-10

**Authors:** Kerui Xu, Shinobu Watanabe-Galloway, Ming Qu, Brandon Grimm, Jungyoon Kim

**Affiliations:** 1 Department of Epidemiology, College of Public Health, University of Nebraska Medical Center, 984395 Nebraska Medical Center, Omaha, NE 68198-4395, US; 2 Division of Public Health, Nebraska Department of Health and Human Services, 301 Centennial Mall South, Lincoln, NE 68509-5026, US; 3 Department of Health Promotion, Social and Behavioral Health, College of Public Health, University of Nebraska Medical Center, 986075 Nebraska Medical Center, Omaha, NE 68198-6075, US; 4 Department of Health Services Research and Administration, College of Public Health, University of Nebraska Medical Center, 984350 Nebraska Medical Center, Omaha, NE 68198-4350, US

## Abstract

**Background:**

According to the U.S. State Department’s Refugee Processing Center and the U.S. Census Bureau, in the fiscal year 2016, among all states in the United States, Nebraska resettled the highest number of refugees per capita.

**Objectives:**

The objectives of this study were to determine the most common reasons for refugees utilizing hospital services in Nebraska between January 2011 and September 2015, and to examine whether refugee patients had increased risks for adverse health conditions compared to non-refugee patients.

**Methods:**

Statewide linkage was performed between Nebraska Medicaid Program’s immigration data, and 2011–2015 Nebraska hospital discharge data inpatient and outpatient files. The linkage produced 3017, 5460, and 775 cases for emergency department visits, outpatient clinic visits, and inpatient care for the refugee sample, respectively.

**Findings:**

Refugee patients were at increased risk for a number of diagnoses or medical conditions, including pregnancy complications, abdominal pain, upper respiratory infections, viral infections, mood disorders, disorders of teeth and jaw, deficiency and anemia, urinary system disorders, headache, nausea and vomiting, limb fractures, spondylosis, essential hypertension, and uncomplicated diabetes mellitus.

**Conclusions:**

The findings suggest a greater emphasis on preventive healthcare, especially in areas of maternal health and perinatal outcomes, psychological counseling, screening for infectious diseases, nutrition and healthy eating, and oral health. Additionally, culturally appropriate measures to address prevention, health screening, and treatments should be adopted by health providers who care for refugees.

## Introduction

The United States has a long history of providing protection and assistance to refugees from all over the world. In response to the large number of refugees coming to the U.S. in the 1970s from Vietnam and Cambodia, the Refugee Act of 1980, which created the Federal Refugee Resettlement Program was established [1]. Between 1980 and June of 2017, over 3 million individuals with a refugee background have been resettled in the U.S. [2]. In spite of this large number of refugees living in the country, there is lack of understanding of health status and health needs of this vulnerable population.

People of refugee background can have significant health care needs due to years of life spent in refugee camps and other settings where limited health services are available [3]. After resettlement to the U.S., cultural, language, and financial constraints may also prevent people of refugee background from having adequate access to health care [4]. Due to their limited awareness of the need for preventive care [5], refugees may utilize emergency room (ER) or other urgent care more frequently compared to the general population. There were some studies conducted to understand the patterns of health care utilization among refugee patients, but most of these studies were based on data from a single health care facility [6,7,8]. Thus, currently, there is dearth of data on the population-level health care utilization patterns among refugee patients. Population-level studies of refugees are difficult because most of the existing data sources do not include information to identify refugee patients. One solution is to conduct a data linkage between a data set which contains immigration or refugee status data with healthcare utilization datasets.

Partly due to relatively low living expense and opportunities for employment, Nebraska has attracted many refugees to resettle. Between 2002 and 2015, approximately 10,000 refugees were newly settled to Nebraska. According to the Pew Research Center’s analysis of data from the U.S. State Department’s Refugee Processing Center and the U.S. Census Bureau, in the fiscal year 2016, 76 refugees were resettled per 100,000 Nebraskan [9]. This indicates that among all U.S. states, Nebraska resettled the highest number of refugees per capita. We linked the immigration database with emergency department, outpatient, and inpatient data files to determine the most common reasons for refugees utilizing hospital services and to compare the risks of being diagnosed with certain conditions between refugee and non-refugee patients.

## Methods

### Data Source

This study utilized the Nebraska hospital discharge data (NHDD) inpatient and outpatient files, which are compiled by the Nebraska Hospital Association (NHA). The outpatient file consists of records of both emergency department and outpatient clinic visits. The information on each hospital discharge is reported from acute care hospitals in Nebraska, and data have been collected annually since 1975. The NHDD include patient-identifiable information, demographic characteristics, disposition information, diagnostic codes, and procedure codes of all hospital visits that occurred at Nebraska’s 87 non-military hospitals. This study also used Nebraska immigration data, which was obtained from the Nebraska Department of Health and Human Services (NDHHS) Medicaid Program. The immigration data contain information of immigration status, date of entry in the U.S., demographics information, address, and Medicaid eligibility information. Records of individuals with immigration status of “Refugee Resettlement Program,” “Refugee-Section 207,” or “Refugee-Employment Authorized” met the eligibility criteria and were identified as refugees.

### Study Populations

The study consists of two groups, including refugee patients and non-refugee patients as referent subjects. To conduct a retrospective cohort study, data of refugee cases recorded by the NDHHS Medicaid Program since it began collection through the end of 2015, and NHDD inpatient and outpatient files that occurred between January 2011 and September 2015 were obtained. In order to acquire hospital information of refugee patients, probabilistic linkage was performed between immigration data and NHDD files. Link Plus Software was utilized, and variables used for linkage included patient name, gender, and date of birth. Manual review and a check on linkage quality was performed. A total record of 3,017 emergency department visits (based on 1,047 refugee patients), 5,460 outpatient clinic visits (1,803 refugee patients), and 775 hospitalizations (558 refugee patients) were found in Nebraska hospital data between January 2011 and September 2015.

The referent subjects were obtained from the NHDD, and consisted of all non-refugee residents who received hospital services in Nebraska between January 2011 and September 2015. To obtain data of non-refugee patients, records of patients with refugee status were subtracted from the total records of patients who received hospital services in Nebraska between January 2011 and September 2015. For the referent subjects, the final count consisted of 1,831,422 emergency department visits (based on 622,405 non-refugee patients), 5,331,679 outpatient clinic visits (1,372,237 non-refugee patients), and 735,037 hospitalizations (469,510 non-refugee patients).

### Statistical Analysis

Descriptive statistics were produced for refugee and non-refugee patients to compare for the study populations’ characteristics. The most commonly diagnosed conditions among refugees who received hospital services between January 2011 and September 2015 were generated. All analyses were based on records of primary diagnosis codes. Diagnoses were coded using the International Classification of Diseases, Ninth Revision, Clinical Modification (ICD-9-CM). The Clinical Classifications Software (CCS) for ICD-9, developed by the Healthcare Cost and Utilization Project, was employed to analyze data on diagnoses [10]. The CCS provides an organized categorization scheme that incorporates 14,000 ICD-9-CM codes and collapses them into a smaller number of clinically meaningful categories, which enables for analysis of broad categories of diagnoses. In this study, to provide a more comprehensive assessment of adverse pregnancy outcomes, CCS categories 177 through 195 were grouped into a larger category labeled as “pregnancy complications.” The relative risks (RR) and 95% confidence intervals (CI) were computed to compare the common reasons for utilizing hospital services between refugee and non-refugee patients, and comparisons were stratified by gender (male and female) and age groups (≤19, 20–39, and ≥40). All analyses were performed separately for outpatient clinic visits, emergency department visits, and hospitalizations. Statistical analyses were conducted using SAS 9.4 (SAS Institute, Inc.), with a two-tailed significance of *P* < .05.

The University of Nebraska Medical Center Institutional Review Board determined this study as exempt.

## Results

### Demographics Characteristics

The age and gender distributions of the overall Nebraska refugee populations were obtained prior to linkage with hospital discharge databases. According to Nebraska Medicaid Program’s immigration data, 16.3%, 28.8%, 18.0%, 14.2%, 14.7%, and 8.1% of resettled refugees belonged to age groups of <10, 10–19, 20–29, 30–39, 40–59, and ≥60, respectively. In terms of gender, 49.3% were males and 50.7% were females. As shown in Table 1, linked data demonstrated that 75.3% of refugees with emergency visits were 39 years old or younger, followed by 54.4% for outpatient visits, and 69.9% for hospitalizations. The majority of refugees using outpatient services (55.7%) and hospitalization services (75.5%) were women. The largest proportion of refugee patients were Asian, and 66.3% of refugees visited emergency department were not married while 60.8% of refugees who were hospitalized were married. In terms of health insurance status, commercial insurance was the most common (56.2%) primary payer among refugees who received emergency services. For refugees with outpatient visits and hospitalizations, the majority listed Medicaid as the primary payer at 58.6% and 55.7%. In contrast, only 7.1% of non-refugee patients used outpatient services and 9.4% of non-refugee patients who were hospitalized listed Medicaid as the primary payer. In addition, while 9.7%, 21.5%, and 34.9% of non-refugee patients with emergency visits, outpatient visits, and hospitalizations, respectively, used Medicare as the primary payer, the corresponding percentages for refugee patients were only 0.9%, 1.1%, and 1.8%, respectively.

**Table 1:** Demographic characteristics and patient information of refugee and non-refugee patients who received hospital services in Nebraska between January 1st, 2011, and September 30th, 2015.

Characteristics	Outpatient clinic visits		Emergency department visits		Hospitalizations	

	Refugee, n (%)	Non-refugee, n (%)	Refugee, n (%)	Non-refugee, n (%)	Refugee, n (%)	Non-refugee, n (%)

Age of visit (years)*						
<10	688 (12.6)	385,287 (7.2)	757 (25.1)	348,284 (19.0)	33 (4.3)	28,421 (3.9)
10–19	636 (11.7)	336,289 (6.3)	615 (20.4)	223,875 (12.2)	83 (10.7)	31,777 (4.3)
20–29	882 (16.2)	425,939 (8.0)	467 (15.5)	304,286 (16.6)	253 (32.7)	94,638 (12.9)
30–39	764 (14.0)	470,422 (8.8)	434 (14.4)	230,584 (12.6)	173 (22.3)	80,183 (10.9)
40–59	1,267 (23.2)	1,433,010 (26.9)	476 (15.8)	365,110 (19.9)	96 (12.4)	149,158 (20.3)
≥60	1,223 (22.4)	2,280,732 (42.8)	268 (8.9)	359,283 (19.6)	137 (17.7)	350,860 (47.7)
Total	5,460	5,331,679	3,017	1,831,422	775	735,037
Gender						
Male	798 (44.3)	568,021 (41.4)	514 (49.1)	322,998 (51.9)	137 (24.6)	179,650 (38.3)
Female	1,005 (55.7)	803,159 (58.5)	533 (50.9)	298,947 (48.1)	421 (75.5)	289,805 (61.7)
Unknown	0 (0.0)	1,057 (0.1)	0 (0.0)	460 (0.1)	0 (0.0)	55 (0.01)
Total	1,803	1,372,237	1,047	622,405	558	469,510
Hospital charge ($)*						
Mean ± SD	1,671.7±4,366.7	1,863.3±5,569.4	2,222.0±4,082.4	2,361.2±3,656.9	31,519.4±80,277.1	32,442.3±53,204.8
Total	16,210,669	20,174,729,619	8,530,750	5,458,345,404	28,367,433	27,840,593,539
Payer source						
Commercial insurance	622 (34.5)	885,387 (64.5)	588 (56.2)	375,196 (60.3)	225 (40.3)	228,718 (48.7)
Medicaid	1,056 (58.6)	97,034 (7.1)	401 (38.3)	60,668 (9.8)	311 (55.7)	43,910 (9.4)
Self-Pay	57 (3.2)	45,453 (3.3)	43 (4.1)	83,058 (13.3)	11 (1.8)	17,662 (3.8)
Medicare	20 (1.1)	295,643 (21.5)	9 (0.9)	60,194 (9.7)	10 (1.8)	163,655 (34.9)
Federal program	48 (2.7)	48,720 (3.6)	6 (0.6)	43,289 (7.0)	1 (0.2)	15,565 (3.3)
Total	1,803	1,372,237	1,047	622,405	558	469,510
Race						
Asian	1,053 (58.4)	****	326 (31.1)	****	272 (48.7)	****
White	241 (13.4)	****	291 (27.8)	****	62 (11.1)	****
African	333 (18.5)	****	257 (24.5)	****	164 (29.4)	****
Other	29 (1.6)	****	32 (3.1)	****	14 (2.5)	****
Unknown	147 (8.2)	****	141 (13.5)	****	46 (8.2)	****
Total	1,803	****	1,047	****	558	****
Marital Status						
Not married	871 (48.3)	****	694 (66.3)	****	183 (32.8)	****
Married	802 (44.5)	****	307 (29.3)	****	339 (60.8)	****
Widowed	106 (5.9)	****	34 (3.3)	****	27 (4.8)	****
Divorced	24 (1.3)	****	12 (1.2)	****	9 (1.6)	****
Total	1,803	****	1,047	****	558	****

* Records-based information. **** Information on race and marital status are incomplete/unavailable from Nebraska hospital discharge databases.

### Comparisons of Refugees and Non-refugees by Gender

The most common reasons for emergency visits, outpatient visits, and hospitalizations among refugee patients compared to non-refugee referent subjects by gender are presented in Tables 2 and 3. As shown in Table 2, pregnancy complications were the top reason for female refugees to receive hospital services. Compared to referent subjects, female refugees with emergency visits, outpatient visits, and hospitalizations had 2.83 (95% CI: 2.43–3.29; *P* < .0001), 2.49 (95% CI: 2.20–2.81; *P* < .0001), and 2.88 (95% CI: 2.70–3.08; *P* < .0001) times higher risk for pregnancy complications, respectively. Furthermore, they were 5.13 (95% CI: 4.53–5.81; *P* < .0001) and 3.48 (95% CI: 2.40–5.03; *P* < .0001) times more likely to have outpatient visits and hospitalizations, respectively, for normal pregnancy/delivery. In comparison, female refugees with emergency visits also had higher risks for diagnoses of headache, urinary tract infections, nausea and vomiting, and disorders of teeth and jaw, while those with outpatient visits were more likely to have abdominal pain and to receive immunizations/screening for infectious disease.

**Table 2:** Comparisons of reasons for hospital visits between female refugee and non-refugee patients who received hospital services in Nebraska between January 1st, 2011, and September 30th, 2015.

Type of hospital visit	Top reasons among refugees	Refugees, n (%)	Non-refugees, n (%)	RR (95% CI)	*P* value	

Outpatient clinic visits						
	1. Pregnancy complications (CCS 177–195)	241 (7.2)	95,454 (2.9)	2.49 (2.20–2.81)	<.0001	*
	2. Normal pregnancy and/or delivery (CCS 196)	232 (6.9)	44,553 (1.3)	5.13 (4.53–5.81)	<.0001	*
	3. Other screening for suspected conditions (not mental disorders or infectious disease) (CCS 258)	229 (6.8)	336,793 (10.2)	0.67 (0.59–0.76)	<.0001	*
	4. Abdominal pain (CCS 251)	146 (4.3)	93,696 (2.8)	1.53 (1.31–1.80)	<.0001	*
	5. Immunizations and screening for infectious disease (CCS 10)	116 (3.4)	26,863 (0.8)	4.25 (3.55–5.09)	<.0001	*
	6. Spondylosis; intervertebral disc disorders; other back problems (CCS 205)	97 (2.9)	80,942 (2.4)	1.18 (0.97–1.44)	0.10	
	7. Medical examination/evaluation (CCS 256)	94 (2.8)	148,381 (4.5)	0.62 (0.51–0.76)	<.0001	*
	8. Non-traumatic joint disorders (CCS 204)	91 (2.7)	94,737 (2.9)	0.95 (0.77–1.16)	.59	
	9. Genitourinary symptoms and ill-defined conditions (CCS 163)	82 (2.4)	66,893 (2.0)	1.21 (0.97–1.50)	.08	
	10. Rehabilitation care; fitting of prostheses; and adjustment of devices (CCS 254)	79 (2.3)	77,915 (2.4)	1.00 (0.80–1.24)	.99	
	Other reasons/conditions	1,963 (58.2)	2,252,575 (67.9)			
	Total	3,370	3,318,802			
Emergency department visits						
	1. Pregnancy complications (CCS 177–195)	154 (9.3)	32,912 (3.3)	2.83 (2.43–3.29)	<.0001	*
	2. Upper respiratory infections (CCS 126)	114 (6.9)	40,239 (4.0)	1.71 (1.43–2.04)	<.0001	*
	3. Abdominal pain (CCS 251)	97 (5.9)	50,512 (5.0)	1.16 (0.96–1.41)	.13	
	4. Headache; including migraine (CCS 84)	77 (4.6)	29,333 (2.9)	1.59 (1.27–1.97)	<.0001	*
	5. Urinary tract infections (CCS 159)	75 (4.5)	29,087 (2.9)	1.56 (1.25–1.94)	<.0001	*
	6. Sprains and strains (CCS 232)	65 (3.9)	45,169 (4.5)	0.87 (0.68–1.10)	.25	
	7. Nausea and vomiting (CCS 250)	55 (3.3)	22,973 (2.3)	1.45 (1.11–1.88)	.006	*
	8. Superficial injury; contusion (CCS 239)	54 (3.3)	51,716 (5.2)	0.63 (0.49–0.82)	<.001	*
	9. Disorders of teeth and jaw (CCS 136)	39 (2.4)	11,235 (1.1)	2.10 (1.54–2.86)	<.0001	*
	10. Spondylosis; intervertebral disc disorders; other back problems (CCS 205)	35 (2.1)	28,408 (2.8)	0.74 (0.54–1.03)	.08	
	Other reasons/conditions	894 (53.9)	660,315 (65.9)			
	Total	1,659	1,001,899			
Hospitalizations						
	1. Pregnancy complications (CCS 177–195)	351 (62.2)	95,631 (21.6)	2.88 (2.70–3.08)	<.0001	*
	2. Normal pregnancy and/or delivery (CCS 196)	27 (4.8)	6,105 (1.4)	3.48 (2.40–5.03)	<.0001	*
	3. Mood disorders (CCS 657)	13 (2.3)	14,037 (3.2)	0.73 (0.43–1.25)	.24	
	4. Septicemia (except in labor) (CCS 2)	9 (1.6)	10,580 (2.4)	0.67 (0.35–1.28)	.22	
	5. Biliary tract disease (CCS 149)	9 (1.6)	5,159 (1.2)	1.37 (0.72–2.62)	.34	
	Other reasons/conditions	155 (27.5)	311,647 (70.3)			
	Total	564	443,159			

* Statistical significance at *P* < .05.

**Table 3:** Comparisons of reasons for hospital visits between male refugee and non-refugee patients who received hospital services in Nebraska between January 1st, 2011, and September 30th, 2015.

Type of hospital visit	Top reasons among refugees	Refugees, n (%)	Non-refugees, n (%)	RR (95% CI)	*P* value	

Outpatient clinic visits						
	1. Immunizations and screening for infectious disease (CCS 10)	113 (5.4)	11,007 (0.6)	9.88 (8.25–11.83)	<.0001	*
	2. Abdominal pain (CCS 251)	88 (4.2)	46,278 (2.3)	1.83 (1.49–2.25)	<.0001	*
	3. Lower respiratory disease (CCS 133)	70 (3.4)	65,321 (3.3)	1.03 (0.82–1.30)	.79	
	4. Administrative/social admissions (CCS 255)	69 (3.3)	22,582 (1.1)	2.94 (2.33–3.71)	<.0001	*
	5. Disorders of teeth and jaw (CCS 136)	64 (3.1)	7,755 (0.4)	7.94 (6.23–10.12)	<.0001	*
	6. Spondylosis; intervertebral disc disorders; other back problems (CCS 205)	64 (3.1)	58,555 (2.9)	1.05 (0.83–1.34)	.68	
	7. Rehabilitation care; fitting of prostheses; and adjustment of devices (CCS 254)	62 (3.0)	60,242 (3.0)	0.99 (0.78–1.27)	.94	
	8. Essential hypertension (CCS 98)	57 (2.7)	55,076 (2.7)	1.00 (0.77–1.29)	.98	
	9. Genitourinary symptoms and ill-defined conditions (CCS 163)	57 (2.7)	30,542 (1.5)	1.80 (1.39–2.32)	<.0001	*
	10. Medical examination/evaluation (CCS 256)	57 (2.7)	88,888 (4.4)	0.62 (0.48–0.80)	<.001	*
	Other reasons/conditions	1,389 (66.5)	1,565,105 (77.8)			
	Total	2,090	2,011,351			
Emergency department visits						
	1. Upper respiratory infections (CCS 126)	101 (7.4)	34,789 (4.2)	1.77 (1.47–2.14)	<.0001	*
	2. Superficial injury; contusion (CCS 239)	91 (6.7)	48,303 (5.8)	1.15 (0.94–1.40)	.17	
	3. Abdominal pain (CCS 251)	61 (4.5)	27,774 (3.4)	1.34 (1.05–1.71)	.02	*
	4. Sprains and strains (CCS 232)	61 (4.5)	35,548 (4.3)	1.05 (0.82–1.34)	.71	
	5. Viral infection (CCS 7)	43 (3.2)	12,274 (1.5)	2.14 (1.59–2.87)	<.0001	*
	6. Nausea and vomiting (CCS 250)	42 (3.1)	14,606 (1.8)	1.76 (1.30–2.37)	< 001	*
	7. Open wounds of extremities (CCS 236)	42 (3.1)	43,832 (5.3)	0.58 (0.43–0.79)	<.001	*
	8. Fracture of upper limb (CCS 229)	41 (3.0)	18,367 (2.2)	1.36 (1.01–1.84)	.045	*
	9. Headache; including migraine (CCS 84)	36 (2.7)	12,562 (1.5)	1.75 (1.27–2.42)	<.001	*
	10. Otitis media and related conditions (CCS 92)	36 (2.7)	16,391 (2.0)	1.34 (0.97–1.85)	.07	
	Other reasons/conditions	804 (59.2)	564,508 (68.1)			
	Total	1,358	828,954			
Hospitalizations						
	1. Mood disorders (CCS 657)	14 (6.6)	10,575 (3.6)	1.83 (1.10–3.04)	.02	*
	2. Acute cerebrovascular disease (CCS 109)	12 (5.7)	6,194 (2.1)	2.68 (1.55–4.64)	.002	*
	3. Schizophrenia and other psychotic disorders (CCS 659)	7 (3.3)	2,762 (0.9)	3.51 (1.69–7.27)	.004	*
	4. Epilepsy; convulsions (CCS 83)	6 (2.8)	2,271 (0.8)	3.65 (1.66–8.05)	.007	*
	5. Fracture of lower limb (CCS 230)	6 (2.8)	2,800 (1.0)	2.96 (1.35–6.53)	.02	*
	Other reasons/conditions	166 (78.7)	267,220 (91.6)			
	Total	211	291,822			

* Statistical significance at *P* < .05.

For male refugee patients (Table 3), the top reason for receiving emergency services was for upper respiratory infections, and male refugee patients had 1.77 (95% CI: 1.47–2.14; *P* < .0001) times higher risk of being diagnosed with upper respiratory infections compared to referent subjects. Moreover, male refugees who used outpatient services were at a significantly higher risk for disorders of teeth and jaw (RR: 7.94; 95% CI: 6.23–10.12; *P* < .0001). In comparison, hospitalized male refugees were more likely to have psychological disorders, as they had 1.83 (95% CI: 1.10–3.04; *P* = .02) and 3.51 (95% CI: 1.69–7.27; *P* = .004) times higher risks for mood disorders and schizophrenia, respectively. Additionally, male refugees with emergency visits had higher risks for diagnoses of abdominal pain, viral infections, nausea and vomiting, fracture of upper limb, and headache. Male refugees who used outpatient services were more likely to have immunizations/screening for infectious disease, administrative or social admissions, abdominal pain, and genitourinary symptoms/conditions, while those hospitalized had higher risks of acute cerebrovascular disease, epilepsy, and fracture of lower limb.

### Comparisons of Refugees and Non-refugees by Age Groups

Comparisons of the most common reasons for utilizing hospital services between refugee and non-refugee patients were also stratified by age groups. According to Appendix A, among refugee patients 19 years old or younger, upper respiratory infections were the top reason to receive emergency services, and compared to referent subjects, refugees aged ≤19 had a slightly higher risk (RR: 1.34; 95% CI: 1.15–1.55; *P* < .001). In comparison, refugees aged ≤19 were at higher risks for both upper and lower limb fractures. Interestingly, outpatient refugees aged ≤19 had significantly higher risks for having a diagnosis of disorders of teeth and jaw (RR: 6.65; 95% CI: 5.60–7.89; *P* < .0001) and deficiency and anemia (RR: 9.53; 95% CI: 7.50–12.10; *P* < .0001). Moreover, refugees aged ≤19 with emergency visits were at higher risks for abdominal pain and nausea and vomiting, while those with outpatient visits were more likely to have nutritional, endocrine, and metabolic disorders and to receive immunizations/screening for infectious disease.

As shown in Appendix B, pregnancy complications were the main reason for emergency visits, outpatient visits, and hospitalizations among female refugees aged 20 to 39. Compared to referent subjects, refugees aged 20–39 with emergency visits, outpatient visits, and hospitalizations had 2.94 (95% CI: 2.52–3.43; *P* < .0001), 1.32 (95% CI: 1.16–1.50; *P* < .0001), and 1.25 (95% CI: 1.18–1.32; *P* < .0001) times higher risk for pregnancy complications, respectively. Urinary system disorders also posed a higher risk, as refugees aged 20–39 with emergency visits were 1.93 (95% CI: 1.42–2.63; *P* < .0001) times more likely to be diagnosed with urinary tract infections and outpatient refugees aged 20–39 were 1.67 (95% CI: 1.25–2.24; *P* < .001) times more likely to have genitourinary symptoms/conditions. Furthermore, in comparison, refugees aged 20–39 with emergency visits were at higher risks for headache and upper respiratory infections. Refugees with outpatient visits were also more likely to have normal pregnancy/delivery and to receive immunizations/screening for infectious disease, and those hospitalized were more likely to have normal pregnancy/delivery and epilepsy.

Compared to referent subjects, outpatient refugees aged 40 or older had higher risks for spondylosis and other back problems (RR: 1.59; 95% CI: 1.33–1.90; *P* < .0001), essential hypertension (RR: 1.34; 95% CI: 1.12–1.61; *P* = .002), abdominal pain (RR: 1.98; 95% CI: 1.65–2.39; *P* < .0001), lower respiratory disease (RR: 1.33; 95% CI: 1.09–1.61; *P* = .005), and uncomplicated diabetes mellitus (RR: 1.79; 95% CI: 1.46–2.20; *P* < .0001) (Appendix C). In addition to those who used outpatient services, refugees aged ≥40 with emergency visits had higher risks for abdominal pain, headache, urinary tract infections, and disorders of teeth and jaw, and those hospitalized were more likely to have pregnancy complications, acute cerebrovascular disease, and biliary tract disease.

## Discussion

To our knowledge, this is the largest study utilizing the linked statewide population-based data from the U.S. to assess the common reasons for refugee populations receiving hospital services. This study found that Nebraska refugee patients were at increased risk for a number of preventable health conditions compared to non-refugee patients, including but not limited to pregnancy complications, abdominal pain, upper respiratory infections, viral infections, psychological disorders, disorders of teeth and jaw, deficiency and anemia, urinary system disorders, headache, nausea and vomiting, upper and lower limb fractures, spondylosis, essential hypertension, and uncomplicated diabetes mellitus.

Pregnancy complications posed higher risks in female refugee patients, especially those between 20 and 29 years old. Consistent with previous findings, a study conducted in Australia has shown that refugee women from various African regions had greater risks of adverse pregnancy outcomes compared to non-refugee migrant women [11]. Unplanned birth before hospital arrival was higher among refugees from North Africa, and West African refugees had the highest stillbirth incidence [11]. A similar study conducted among Asian refugees in Australia found that women from Afghanistan, Iraq, Bhutan, and Myanmar had poorer maternal health in general, and those from South Asia had increased risks of post-term birth and lower engagement in prenatal care [12]. Furthermore, refugees from Sub-Saharan Africa who gave birth in Toronto exhibited significantly higher rates of delivery by caesarean section and having low birth weight infants compared to non-refugees [13]. A review on maternal health of immigrant women in Canada found that prenatal care in uninsured asylum-seeking refugees was inadequate, and severe disparities in maternal morbidity were present [14].

In addition to pregnancy complications, abdominal pain was a common reason among refugees utilizing outpatient clinic and emergency department services. Van Berlaer et al. [6] examined the clinical characteristics of 3,907 refugees resettled in Belgium, and observed that abdominal pain was among the most common symptoms. Abdominal pain may indicate a parasitic or bacterial infection, including the *Helicobacter pylori* (*H. Pylori*) infection. It is known that refugees who resettle in western countries frequently present with high rates of *H. pylori* infection, and over 80% of African refugee children in Australia have a positive stool antigen test upon arrival [15]. Thus, refugee patients presenting with abdominal pain are frequently assessed for intestinal parasites [16], and screening for *H. pylori* in populations with high prevalence has been suggested to be cost-effective [15].

Since refugees are at risk for a variety of infectious diseases that may be rare in the U.S., extensive screening procedures are often involved [17], which is demonstrated in our results that refugee patients were more likely to receive immunizations/screening of infectious disease. Furthermore, upper respiratory infections were the top reason for refugees aged 19 or younger to utilize emergency department visits. Similar to our results, respiratory tract infection was the most commonly diagnosed illness among child refugees who received emergency services in a hospital in Lesbos, Greece [7]. In addition, the World Health Organization has reported core concerns of upper respiratory infections for resettled refugees [18], and analyses of data from 90 United Nations High Commissioner for Refugees camps of 16 countries from Asia and Africa indicated that upper respiratory infections accounted for 29% of morbidity in children under the age of five [19].

Psychiatric disorders have been well-recognized among refugee populations, and a number of mental disorders are caused by violence, war, torture, exile, and uncertainty of status in foreign countries [20]. In this study, we observed that hospitalized male refugees were more likely to have mood disorders. Correspondingly, many studies have found high levels of distress, depression, and post-traumatic disorders among various refugee populations who resettled in western countries, including the U.S. and Canada [20,21,22,23]. In addition to mood disorders, meta-analytic reviews have found increased risks of schizophrenia and psychosis among migrant populations, and the risk is even higher for second-generation migrants [24,25]. Similarly, in this study, the risk for having a diagnosis of schizophrenia was elevated among hospitalized male refugees. Factors such as discrimination upon arrival, acceptance into the community, financial issues, health literacy, language and cultural barriers, and previous trauma may jointly contribute to the development of psychological disorders among resettled refugees [26].

Our results indicated that young refugees with outpatient clinic services were at significantly higher risks for disorders of teeth and jaw, and deficiency and anemia. It is known that many refugee children have never been exposed to basic preventive oral care [27]. Refugee oral health assessments conducted at the Massachusetts Department of Public Health found that white refugee children were three times more likely to have caries compared to white or African American non-refugee children, and were 9.4 times more likely to have untreated caries [27]. A review of 32 studies that examined oral health status of immigrant and refugee children in North America found that migrant children suffered from poor oral health and were facing a variety of barriers to receive dental care services [28]. In terms of anemia, a retrospective chart review of refugees born in a clinic in Toronto observed high rates of anemia (22.8%) and iron deficiency (53.3%) [29]. Moreover, 30% of resettled Bhutanese refugees in the U.S. were found to be vitamin B12 deficient in a study conducted by the Centers for Disease Control and Prevention [30], and prevalence of anemia was observed at over 40% among refugee children aged 6–59 months who resettled in the U.S. [31]. Studies have also found a high prevalence of iron-deficiency anemia among children from refugee camps in North and East Africa regions, as well as in Kenya, Nepal, and Burma [32,33,34].

The major strengths of this study are that findings were based from record linkage between large statewide databases, and the large sample size enables of generating statistically meaningful findings. Nevertheless, this study is subject to certain limitations. Using relative risk as a measurement, comparisons of the common reasons for receiving hospital services between refugees and non-refugees were limited to hospital patients only, rather than the general populations who resided in Nebraska. Therefore, the findings that refugees have an increased risk for certain conditions are based on hospital patients, and may not be generalizable to refugees who are not seen or treated in hospitals. Additionally, in the immigration data, information on “country of citizenship” and “country of birth” were largely missing. Since refugees are a heterogeneous group with diversity in various aspects, there is a need to examine refugees from similar regions or ethnic groups. This limitation calls for an improvement in data collection, and an emphasis to accurately capture the nationality of refugees. Lastly, infants born of refugee mothers were not contained in the immigration data due to their American citizenship status.

## Conclusions and future directions

Our findings suggest a greater focus on preventive healthcare and public health interventions, especially in areas of maternal health and perinatal outcomes, psychological counseling, screening for infectious diseases, nutrition and healthy eating, and oral and dental care. Refugees resettled in the U.S. arrive from different countries and cultures, and they often require complex health care needs upon arrival. As vulnerable populations, even after resettlement, many refugees face continued struggles in poverty, discrimination, language barriers, and unfamiliarity with the local environment and health care systems [35]. Outreach programs that prioritize all refugees are needed. Refugee support groups should work with community health workers, local and state public health departments, local non-profits, and primary health providers to identify the at-risk individuals, and implement effective preventive measures for early disease detection and educational counseling on living a healthy lifestyle.

Findings from this study indicate a more coordinated effort to provide primary care services to refugees. Refugees often come from countries where primary care does not exist or is not well-established. In our findings, the majority of adverse health conditions presented among refugees could be prevented or managed through primary care. All refugees are required to receive the refugee health assessment after arriving in the U.S. [36]; however, the assessment is often conducted by agencies that are not primary care providers [37]. Although refugees may be eligible for Medicaid, the initial coverage expires after six months of living in the U.S. During this period, refugees are busy finding schools for their children, going through job training, taking English classes, and looking for jobs. Even when they have an employee health insurance, they are unlikely to seek routine physical exams or care from primary care providers because they are not familiar with disease prevention concepts or practice. In Nebraska and other states, a refugee health navigator program has been piloted to help refugees to get connected with primary care and to navigate a complex health care system in the U.S. Additionally, since there is no universal coverage of basic health insurance in the U.S., access to preventive primary care is particularly important for resettled immigrant and refugee populations [38]. Primary care provides a greater access to care for relatively deprived populations, better quality of care, a greater emphasis on prevention, early disease management, and reduction for unnecessary and potentially harmful specialist care [39]. Furthermore, language and miscommunication between health providers and refugee patients have been reported to be the main barriers that deter access to quality healthcare [40,41]. Therefore, interventions should focus on issues that include language barriers, cultural differences, and poor health literacy. Culturally appropriate measures to address prevention, health screening, and treatments should be adopted by health providers who provide care for refugees.

## Additional Files

The additional files for this article can be found as follows:

10.29024/aogh.2354.s1 Appendix A. Comparisons of reasons for hospital visits between refugee and non-refugee patients aged 19 or younger who received hospital services in Nebraska between January 1st, 2011, and September 30th, 2015.

10.29024/aogh.2354.s1 Appendix B. Comparisons of reasons for hospital visits between refugee and non-refugee patients aged 20 to 39 who received hospital services in Nebraska between January 1st, 2011, and September 30th, 2015.

10.29024/aogh.2354.s1 Appendix C. Comparisons of reasons for hospital visits between refugee and non-refugee patients aged 40 or older who received hospital services in Nebraska between January 1st, 2011, and September 30th, 2015.
