# Supplementary material for: Common Diagnoses among Refugee Populations: Linked Results with Statewide Hospital Discharge Database
Source: Ann Glob Health. 2018 Oct 10;84(3):541–50. doi: 10.29024/aogh.2354 (PMC6748192; doi:10.29024/aogh.2354)
Supplement: Appendix A. — Comparisons of reasons for hospital visits between refugee and non-refugee patients aged 19 or younger who received hospital services in Nebraska between January 1st, 2011, and September 30th, 2015. [file agh-84-3-2354-s1.pdf]

Supplemental Materials

Appendix A. Comparisons of reasons for hospital visits between refugee and non-refugee patients aged 19 or younger who received hospital services in Nebraska between January 1st, 2011, and September 30th, 2015.

Type of hospital visit	Top reasons among refugees	Refugees, n (%)	Non-refugees, n (%)	RR (95% CI)	*P* value
Outpatient clinic visits					
	1. Disorders of teeth and jaw (CCS 136)	121 (9.1)	9,919 (1.4)	6.65 (5.60–7.89)	< .0001*
	2. Administrative/social admissions (CCS 255)	112 (8.5)	35,352 (4.9)	1.73 (1.45–2.06)	< .0001*
	3. Deficiency and other anemia (CCS 59)	65 (4.9)	3,718 (0.5)	9.53 (7.50–12.10)	< .0001*
	4. Immunizations and screening for infectious disease (CCS 10)	60 (4.5)	8,134 (1.1)	4.02 (3.14–5.15)	< .0001*
	5. Upper respiratory infections (CCS 126)	59 (4.5)	42,434 (5.9)	0.76 (0.59–0.97)	.03*
	6. Abdominal pain (CCS 251)	51 (3.9)	24,905 (3.5)	1.12 (0.85–1.46)	.43
	7. Genitourinary symptoms and ill-defined conditions (CCS 163)	41 (3.1)	16,723 (2.3)	1.34 (0.99–1.81)	.06
	8. Other nutritional; endocrine; and metabolic disorders (CCS 58)	36 (2.7)	13,250 (1.8)	1.48 (1.07–2.04)	.02*
	9. Non-traumatic joint disorders (CCS 204)	33 (2.5)	27,311 (3.8)	0.66 (0.47–0.92)	.01*
	10. Medical examination/evaluation (CCS 256)	32 (2.4)	14,614 (2.0)	1.19 (0.85–1.68)	.31
	Other reasons/conditions	714 (53.9)	525,216 (72.8)		
	Total	1,324	721,576		
Emergency department visits					
	1. Upper respiratory infections (CCS 126)	151 (11.0)	47,153 (8.2)	1.34 (1.15–1.55)	< .001*
	2. Superficial injury; contusion (CCS 239)	87 (6.3)	39,957 (7.0)	0.91 (0.74–1.11)	.35
	3. Abdominal pain (CCS 251)	64 (4.7)	17,997 (3.2)	1.48 (1.17–1.88)	.001*
	4. Nausea and vomiting (CCS 250)	61 (4.5)	15,160 (2.7)	1.68 (1.31–2.15)	< .0001*
	5. Otitis media and related conditions (CCS 92)	58 (4.2)	27,286 (4.8)	0.89 (0.69–1.14)	.35
	6. Sprains and strains (CCS 232)	57 (4.2)	22,027 (3.9)	1.08 (0.84–1.39)	.56
	7. Viral infection (CCS 7)	53 (3.9)	17,428 (3.1)	1.27 (0.97–1.65)	.08
	8. Fracture of upper limb (CCS 229)	51 (3.7)	15,848 (2.8)	1.34 (1.02–1.76)	.03*
	9. Fever of unknown origin (CCS 246)	49 (3.6)	20,721 (3.6)	0.99 (0.75–1.30)	.92
	10. Open wounds of extremities (CCS 236)	44 (3.2)	19,676 (3.4)	0.93 (0.70–1.25)	.64
	Other reasons/conditions	697 (50.8)	328,906 (57.5)		
	Total	1,372	572,159		
Hospitalizations					
	1. Pregnancy complications (CCS 177–195)	20 (17.2)	7,173 (11.9)	1.45 (0.97–2.16)	.08
	2. Mood disorders (CCS 657)	17 (14.7)	8,120 (13.5)	1.09 (0.70–1.69)	.71
	3. Fracture of lower limb (CCS 230)	5 (4.3)	622 (1.0)	4.17 (1.76–9.87)	.008*
	Other reasons/conditions	74 (63.8)	44,283 (73.6)		
	Total	116	60,198		

*Statistical significance at $P < .05$

Appendix B. Comparisons of reasons for hospital visits between refugee and non-refugee patients aged 20 to 39 who received hospital services in Nebraska between January 1st, 2011, and September 30th, 2015.

Type of hospital visit	Top reasons among refugees	Refugees, n (%)	Non-refugees, n (%)	RR (95% CI)	*P* value
Outpatient clinic visits					
	1. Pregnancy complications (CCS 177–195)	205 (12.5)	84,867 (9.5)	1.32 (1.16–1.50)	< .0001*
	2. Normal pregnancy and/or delivery (CCS 196)	203 (12.3)	39,127 (4.4)	2.83 (2.48–3.21)	< .0001*
	3. Immunizations and screening for infectious disease (CCS 10)	108 (6.6)	18,003 (2.0)	3.27 (2.72–3.92)	< .0001*
	4. Other screening for suspected conditions (not mental disorders or infectious disease) (CCS 258)	88 (5.4)	59,885 (6.7)	0.80 (0.65–0.98)	.03*
	5. Abdominal pain (CCS 251)	77 (4.7)	35,414 (4.0)	1.18 (0.95–1.47)	.13
	6. Medical examination/evaluation (CCS 256)	53 (3.2)	57,129 (6.4)	0.51 (0.39–0.66)	< .0001*
	7. Other female genital disorders (CCS 175)	45 (2.7)	21,553 (2.4)	1.14 (0.85–1.52)	.38
	8. Genitourinary symptoms and ill-defined conditions (CCS 163)	44 (2.7)	14,320 (1.6)	1.67 (1.25–2.24)	< .001*
	9. Rehabilitation care; fitting of prostheses; and adjustment of devices (CCS 254)	37 (2.3)	20,587 (2.3)	0.98 (0.71–1.35)	.89
	9. Spondylosis; intervertebral disc disorders; other back problems (CCS 205)	37 (2.3)	23,923 (2.7)	0.84 (0.61–1.16)	.29
	Other reasons/conditions	749 (45.5)	521,553 (58.2)		
	Total	1,646	896,361		
Emergency department visits					
	1. Pregnancy complications (CCS 177–195)	138 (15.3)	27,860 (5.2)	2.94 (2.52–3.43)	< .0001*
	2. Headache; including migraine (CCS 84)	55 (6.1)	18,838 (3.5)	1.73 (1.34–2.24)	< .0001*
	3. Abdominal pain (CCS 251)	52 (5.8)	31,474 (5.9)	0.98 (0.75–1.28)	.89
	4. Upper respiratory infections (CCS 126)	49 (5.4)	16,717 (3.1)	1.74 (1.32–2.29)	< .0001*
	5. Urinary tract infections (CCS 159)	39 (4.3)	11,972 (2.2)	1.93 (1.42–2.63)	< .0001*
	6. Sprains and strains (CCS 232)	36 (4.0)	30,003 (5.6)	0.71 (0.52–0.98)	.04*
	7. Spondylosis; intervertebral disc disorders; other back problems (CCS 205)	32 (3.6)	19,266 (3.6)	0.99 (0.70–1.39)	.94
	7. Superficial injury; contusion (CCS 239)	32 (3.6)	24,421 (4.6)	0.78 (0.55–1.09)	.15
	9. Nausea and vomiting (CCS 250)	24 (2.7)	11,159 (2.1)	1.28 (0.86–1.90)	.23
	10. Disorders of teeth and jaw (CCS 136)	23 (2.6)	12,536 (2.3)	1.09 (0.73–1.63)	.68
	Other reasons/conditions	421 (46.7)	330,624 (61.8)		
	Total	901	534,870		
Hospitalizations					
	1. Pregnancy complications (CCS 177–195)	315 (73.9)	103,289 (59.1)	1.25 (1.18–1.32)	< .0001*
	2. Normal pregnancy and/or delivery (CCS 196)	26 (6.1)	5,740 (3.3)	1.86 (1.28–2.70)	.001*
	3. Epilepsy; convulsions (CCS 83)	7 (1.6)	1,196 (0.7)	2.40 (1.15–5.02)	.03*
	3. Mood disorders (CCS 657)	7 (1.6)	8,414 (4.8)	0.34 (1.16–0.71)	<.001*
	5. Schizophrenia and other psychotic disorders (CCS 659)	5 (1.2)	1,953 (1.1)	1.05 (0.44–2.51)	.82
	Other reasons/conditions	66 (15.5)	54,229 (31.0)		
	Total	426	174,821		

*Statistical significance at *P* <.05

Appendix C. Comparisons of reasons for hospital visits between refugee and non-refugee patients aged 40 or older who received hospital services in Nebraska between January 1st, 2011, and September 30th, 2015.

Type of hospital visit	Top reasons among refugees	Refugees, n (%)	Non-refugees, n (%)	RR (95% CI)	*P* value
Outpatient clinic visits					
	1. Other screening for suspected conditions (not mental disorders or infectious disease) (CCS 258)	170 (6.8)	315,376 (8.5)	0.80 (0.70–0.93)	.003*
	2. Spondylosis; intervertebral disc disorders; other back problems (CCS 205)	114 (4.6)	106,985 (2.9)	1.59 (1.33–1.90)	< .0001*
	3. Essential hypertension (CCS 98)	109 (4.4)	121,087 (3.3)	1.34 (1.12–1.61)	.002*
	4. Abdominal pain (CCS 251)	106 (4.3)	79,709 (2.2)	1.98 (1.65–2.39)	< .0001*
	5. Other lower respiratory disease (CCS 133)	95 (3.8)	106,922 (2.9)	1.33 (1.09–1.61)	.005*
	6. Diabetes mellitus without complication (CCS 49)	90 (3.6)	74,840 (2.0)	1.79 (1.46–2.20)	< .0001*
	7. Other non-traumatic joint disorders (CCS 204)	81 (3.3)	109,888 (3.0)	1.10 (0.89–1.36)	.39
	7. Rehabilitation care; fitting of prostheses; and adjustment of devices (CCS 254)	81 (3.3)	100,163 (2.7)	1.21 (0.97–1.49)	.09
	9. Medical examination/evaluation (CCS 256)	66 (2.7)	165,595 (4.5)	0.59 (0.47–0.75)	< .0001*
	10. Disorders of lipid metabolism (CCS 53)	63 (2.5)	98,189 (2.6)	0.96 (0.75–1.22)	.72
	Other reasons/conditions	1,515 (60.8)	2,434,988 (65.6)		
	Total	2,490	3,713,742		
Emergency department visits					
	1. Abdominal pain (CCS 251)	42 (5.7)	28,853 (4.0)	1.42 (1.06–1.90)	.02*
	2. Headache; including migraine (CCS 84)	36 (4.8)	15,876 (2.2)	2.21 (1.60–3.04)	< .0001*
	3. Nonspecific chest pain (CCS 102)	33 (4.4)	43,087 (6.0)	0.75 (0.53–1.04)	.08
	3. Sprains and strains (CCS 232)	33 (4.4)	28,700 (4.0)	1.12 (0.80–1.56)	.51
	5. Superficial injury; contusion (CCS 239)	26 (3.5)	35,676 (4.9)	0.71 (0.49–1.04)	.07
	5. Urinary tract infections (CCS 159)	26 (3.5)	14,783 (2.0)	1.71 (1.17–2.50)	.005*
	7. Spondylosis; intervertebral disc disorders; other back problems (CCS 205)	25 (3.4)	25,967 (3.6)	0.94 (0.64–1.38)	.74
	8. Conditions associated with dizziness or vertigo (CCS 93)	21 (2.8)	16,509 (2.3)	1.24 (0.81–1.89)	.32
	9. Disorders of teeth and jaw (CCS 136)	18 (2.4)	5,964 (0.8)	2.94 (1.86–4.64)	< .0001*
	10. Other lower respiratory disease (CCS 133)	17 (2.3)	18,704 (2.6)	0.88 (0.55–1.42)	.61
	Other reasons/conditions	467 (62.8)	490,274 (67.7)		
	Total	744	724,393		
Hospitalizations					
	1. Pregnancy complications (CCS 177–195)	16 (6.9)	2,539 (0.5)	13.52 (8.41–21.73)	< .0001*
	2. Acute cerebrovascular disease (CCS 109)	13 (5.6)	12,164 (2.4)	2.29 (1.35–3.89)	.002*
	3. Septicemia (except in labor) (CCS 2)	11 (4.7)	18,958 (3.8)	1.25 (0.70–2.22)	.46
	4. Biliary tract disease (CCS 149)	9 (3.9)	6,889 (1.4)	2.80 (1.48–5.32)	.006*
	4. Pneumonia (except that caused by tuberculosis or STDs) (CCS 122)	9 (3.9)	20,950 (4.2)	0.92 (0.49–1.75)	.80
	Other reasons/conditions	175 (75.1)	438,518 (87.7)		
	Total	233	500,018		

*Statistical significance at *P* <.05
